# Immune-related adverse effects of checkpoint immunotherapy and implications for the treatment of patients with cancer and autoimmune diseases

**DOI:** 10.3389/fimmu.2023.1197364

**Published:** 2023-06-05

**Authors:** Betul Ibis, Konstantinos Aliazis, Carol Cao, Sasitorn Yenyuwadee, Vassiliki A. Boussiotis

**Affiliations:** ^1^ Division of Hematology-Oncology Beth Israel Deaconess Medical Center, Boston, MA, United States; ^2^ Department of Medicine, Beth Israel Deaconess Medical Center, Boston, MA, United States; ^3^ Harvard College, Cambridge, MA, United States; ^4^ Cancer Center, Beth Israel Deaconess Medical Center, Harvard Medical School, Boston, MA, United States

**Keywords:** Immune checkpoint inhibitors (ICIs), immune-related adverse events (irAEs), immunotherapy, autoimmune disease, cancer

## Abstract

During the past decade, there has been a revolution in cancer therapeutics by the emergence of antibody-based immunotherapies that modulate immune responses against tumors. These therapies have offered treatment options to patients who are no longer responding to classic anti-cancer therapies. By blocking inhibitory signals mediated by surface receptors that are naturally upregulated during activation of antigen-presenting cells (APC) and T cells, predominantly PD-1 and its ligand PD-L1, as well as CTLA-4, such blocking agents have revolutionized cancer treatment. However, breaking these inhibitory signals cannot be selectively targeted to the tumor microenvironment (TME). Since the physiologic role of these inhibitory receptors, known as immune checkpoints (IC) is to maintain peripheral tolerance by preventing the activation of autoreactive immune cells, IC inhibitors (ICI) induce multiple types of immune-related adverse effects (irAEs). These irAEs, together with the natural properties of ICs as gatekeepers of self-tolerance, have precluded the use of ICI in patients with pre-existing autoimmune diseases (ADs). However, currently accumulating data indicates that ICI might be safely administered to such patients. In this review, we discuss mechanisms of well established and newly recognized irAEs and evolving knowledge from the application of ICI therapies in patients with cancer and pre-existing ADs.

## Cancer immunosurveillance and immune escape in the tumor microenvironment

The tumor microenvironment (TME) is a complex multicellular space in which cancer can be subjected to immune-mediated control during the early stages of its evolution, such as the eradication and equilibrium phase, but ultimately favors the development of cancer immune escape by fostering cancer immunoediting. During that stage, under continuous immune pressure, cancer cells develop alterations to overcome the immune attack, resulting in tumors that are resistant to the physiological mechanisms utilized by innate immune cells to recognize and present antigens while shutting down the activation of helper and effector T cells (1).

Tumor-promoting factors in the TME include defective antigen presentation by antigen-presenting cells (APC), mainly dendritic cells (DC), which express inhibitory ligands, upregulation of multiple inhibitory receptors during persistent activation of T cells, such as PD-1, LAG-3, TIGIT, TIM-3, expansion of cell populations that mediate T cell inhibitory functions such as myeloid-derived suppressor cells (MDSCs) and T regulatory (Treg) cells, generation of tumor associated macrophages (TAMs) that lose the ability to phagocytose and present antigens but instead acquire protumorigenic functions, and soluble factors such as IDO, VEGF, and TGF-β, which support cancer cell survival and tumor growth while suppressing the function of immune cells (2).

## Expression of immune checkpoints in the TME

T cells of the TME are critical determinants of tumor containment and progression. During the phase of cancer escape from immune control, T cells that can recognize tumor-associated antigens lose the ability to control tumor growth due to mechanisms of tumor-induced tolerance and immunosuppression. These dysfunctional T cells are characterized by features of T cell exhaustion (T_EX_) similar to those in chronic viral infections, including high expression of ICs including CTLA-4, PD-1, TIM-3, TIGIT and LAG-3, loss of expansion capacity, and impaired effector function as determined by the diminished production of cytokines such as IFN-γ and TNF-α (3). Conceptually, a central goal of novel immunotherapies is to achieve re-invigoration of tumor-specific T_EX_ cells, for which the state of T cell exhaustion might be still reversible, and blockade of ICs has been the main approach to achieve this goal (4, 5).

The co-inhibitory molecules, such as CTLA-4 (CD152) and PD-1 (CD279), are induced during physiologic T cell activation. CTLA-4 being upregulated and acting early during T cell activation, is the high affinity receptor of CD80/CD86. CTLA-4 directly competes with CD28 for binding on CD80/CD86, but also induces depletion of CD80/CD86 by trans-endocytosis (6). Importantly, this interaction simultaneously releases free PD-L1 by eliminating availability of CD80 for PD-L1 engagement in PD-L1: CD80 interaction *in cis* (7). Thus, the interactions of CTLA4 interfere both with the key costimulatory pathway CD80/CD28 and the key co-inhibitory pathway PD-1/PD-L1.

PD-1 is physiologically induced upon TCR-mediated T cell activation. Engagement of PD-1 by its ligands PD-L1 (B7-H1 or CD274) and/or PD-L2 (B7-DC or B7-H3) counteracts TCR signaling and CD28-mediated co-stimulation (8–10). The expression of PD-L1 on tumor cells has served as a biomarker for patient stratification for anti-PD-1 ICI therapy. However, the relative contribution of PD-L1 on tumor cells and other cell types in limiting anti-tumor responses in the TME remains under investigation. By genetic deletion of PD-L1 in tumor cells or innate immune cells of host mice, it was found that PD-L1 expression on each of these compartments equally contributes to immune suppression (11). Subsequently, it was determined that PD-L1 expressed in APC, particularly DC, has the key and causative role in compromising anti-tumor T cell responses (12, 13).

Parallel studies revealed that PD-1 expression in myeloid cells has an important role in lineage fate commitment, effector differentiation and antigen presenting function (14). Specifically, it was found that PD-1 is expressed predominantly in myeloid progenitors, whereas ablation of PD-1 expression resulted their differentiation into mature myeloid cells with predominant features of monocyte and DC differentiation (14). RNAseq studies showed that PD-1 ablation in myeloid cells resulted in the differentiation of tumor infiltrating macrophages with features of potent immune function including activation, differentiation, phagocytosis and enhanced signaling and metabolic programs (15). Consistent with these findings, PD-1 expression in TAMs was associated with diminished phagocytosis and enhanced tumor growth (16). Thus, therapeutic targeting with PD-1/PD-L1 blocking compounds might lead to proinflammatory activation of both T cells and myeloid cells and release of multiple proinflammatory cytokines from both immune compartments.

PD-1 and CTLA-4 are the prototype ICs and the most extensively utilized therapeutic targets in cancer immunotherapy. However, other ICs, such as LAG-3, TIM-3, TIGIT, GITR, or VISTA are also exploited by tumor cells, contributing to the generation of an immunosuppressive TME and escape of immunosurveillance (17). Because clinical experience with ICIs for these receptors is limited, irAEs induced by therapies blocking these ICs have not been well-characterized. For these reasons, in the present review, we will focus on irAEs induced by blockade of CTLA-4 and the PD-1/PD-L1 pathway.

## Role of ICs in central and peripheral tolerance and lessons from genetic models

### CTLA-4

The importance of the CTLA-4 receptor in the establishment of peripheral tolerance was identified early by studies with CTLA-4-deficient mice. These mice developed splenomegaly and lymphadenopathy which led to death within 3-4 weeks of age (18–20). This extensive spontaneous lymphoproliferation resulted in massive infiltration of mononuclear cells in the heart, pancreas, liver, lungs, and other organs causing extensive damage to these tissues (18, 19). T cells from the spleen and lymph nodes showed an activated phenotype with upregulation of activation markers like CD69, CD25, and CD44 (18–20). Depletion of CD4^+^ but not CD8^+^ T cells led to inhibition of immune infiltration into non-lymphoid organs (20). Administration of CTLA-4-Ig to CTLA-4-deficient mice prevented the T cell expansion. When CTLA-4 was deleted in adulthood using a conditional knockout approach, these mice still developed lymphoproliferation and immune infiltration in various organs, including pancreatic β-islets, lungs, and stomach (21). Deletion of CTLA-4 in these adult mice resulted in expansion of Treg in the blood, spleen, and lymph nodes, although the levels of Treg decreased rapidly in the blood but not in the organs. CTLA-4 deletion also led to activation of T conventional cells (Tconv, non-Treg cells). In the collagen-induced arthritis (CIA) model, loss of CTLA-4 promoted a stark exacerbation of the disease with extended damage to the joints (21).

Specific deletion of CTLA-4 from Treg also led to systemic lymphoproliferation and death of the mice, albeit with slower kinetics (22). These mice developed immune infiltration of the myocardium and destruction of myocytes, which the authors speculated it caused heart failure due to myocarditis. When adoptively transferred into T cell-deficient mice, CTLA-4^-/-^ Treg could provide tumor protection, in contrast to CTLA-4^+/+^ Treg, indicating that CTLA4-deficient Treg did not have suppressor function but rather acted as T effector cells. Similarly, in an autoimmune model for diabetes, adoptively transferred CTLA-4-deficient Treg were unable to prevent the destruction of the pancreas and the induction of diabetes (23). In contrast to previous reports which described the development of autoimmunity and exacerbation of autoimmune diseases by CTLA-4 deficiency, subsequent studies showed that CTLA-4 ablation in adult mice resulted in complete or transient resistance to experimental autoimmune encephalomyelitis (EAE), which was interpreted as a consequence of increased expansion of thymic Treg as a result of CTLA-4 deletion (24, 25).

B cells also showed an increased activation profile in CTLA-4^-/-^ mice with upregulation of CD86, Fas, and CD5 but not CD80 (19). This correlated with a striking increase of all Ig subtypes in the serum of CTLA-4-deficient mice (19). In this model, CD4^+^ T cell depletion or administration of CTLA-4-Ig prevented the activation and expansion of B cells (20). CTLA-4 is expressed solely in B-1a B cells which are generated during fetal development (26). Deletion of CTLA-4 from B-1a B cells in CTLA-4^f/f^CD19^Cre/+^ mice led to activation and differentiation of these cells into antigen-presenting cells, and spontaneous germinal center formation in the spleens (26). These mice developed autoantibodies and late autoimmune characteristics which shared features with some human autoimmune diseases. Thus, abrogating CTLA-4 function induces autoimmunity by several mechanisms and by targeting several lymphocyte subsets.

The role of CTLA-4 in maintaining tolerance and preventing development of autoimmunity has also been documented in studies where treatment of mice with anti-CTLA-4 monoclonal antibody (mAb) exacerbated autoimmune diseases. When CTLA-4 was blocked in the mouse model of EAE, there was a marked increase in the proinflammatory cytokines TNF-α, IFN-γ, and IL-2, and symptoms were exacerbated (27, 28). Administration of anti-CTLA-4 blocking mAb also resulted in increased inflammatory foci in the brain and spinal cord, and increased demyelinating lesions (28). Similar results of disease exacerbation were observed using the nonobese diabetic (NOD) mouse model, in which administration of anti-CTLA-4 mAb induced rapid disease onset of about 15 days compared to the usual 5-6 months (29). CD4^+^ T cells from mAb-treated mice displayed a chronically activated phenotypic profile with increased expression levels of CD44 but low levels of CD62L, with a few T cells expressing early activation markers like CD69 and CD25 (29). Deletion of CTLA-4 on Treg led to spontaneous germinal center formation in the spleen and lymph nodes (30).

The *CTLA4* locus has been involved in the regulation of several autoimmune diseases in humans (31). Polymorphisms on *CTLA4* gene have been implicated in diabetes and thyroid disease (32, 33), rheumatoid arthritis (34), primary biliary cholangitis (35), and spontaneous abortion (36). Furthermore, a soluble form of CTLA-4 is present in patients with various autoimmune diseases, such as autoimmune thyroid diseases (37, 38), myasthenia gravis (39), and systemic lupus erythematosus (SLE) (40).

### PD-1

Deletion of PD-1 in mice did not induce an immediate extreme phenotype, such as CTLA-4 deletion, however, in later stages of their lives, PD-1-deficient mice developed autoimmune symptoms. C57BL/6 PD-1^-/-^ mice showed signs of mild splenomegaly early on but appeared to be healthy (41). The number of B cells and myeloid cells in the spleen increased, however, the number of T cells remained stable. An increase of IgA, IgG2b, and IgG3 was present in the serum. At 6 months of age, a few of these mice started showing signs of lupus-like glomerulonephritis and arthritis in the foot joints (42). At 14 months of age, the severity of glomerulonephritis had increased, whereas wild-type mice showed only mild symptoms (42). The severity of arthritis also progressed to an advanced stage in all the PD-1 KO mice.

Because C57BL/6/PD-1^-/-^ mice showed a lupus-like phenotype, C57BL/6 PD-1^-/-^ mice were crossed with the B6 *lpr/lpr* mouse strain, which is used as a model for SLE (43, 44). Glomerulonephritis was present much earlier in the C57BL/6-*lpr/lpr*-PD-1^-/-^ mice compared to C57BL/6*lpr/lpr* mice (42). Depositions of IgG3 and C3 complement were present in the kidneys and histology showed arthritic lesions in the joints much earlier than in the control B6 *lpr/lpr* mice. Lymphadenopathy and extensive hyperplasia of bone marrow were also detected. The severity of symptoms in the C57BL/6*lpr/lpr*-PD-1^-/-^ mice resembled that of the MRL-*FAS^lpr/lpr^* mouse, which develops lupus-like symptoms much earlier than the C57BL/6*lpr/lpr.* PD-1 deletion in the MRL mouse resulted in the development of myocarditis and death of the mice by week 10 (45). Extensive immune infiltration was present in the hearts of the PD-1-deficient mice with both populations of CD4^+^ and CD8^+^ T cells showing an activated phenotype. Autoantibodies against cardiac myosin were also present despite the small number of B cells accumulated in the heart of the MRL PD-1-deficient mice. Notably, an increased accumulation of Mac1^+^Gr1^+^ cells was also present in the heart. These cells were able to strongly suppress infiltrating T cells and were considered similar to myeloid-derived suppressor cells (45).

Deletion of PD-1 in BALB/c mice resulted in a more severe phenotype than in the B57BL/6 mice, with development of multisystem autoimmunity. These mice suffered from splenomegaly, hepatomegaly, cardiomegaly, and immune infiltration of several organs such as the heart, lungs, liver, kidney, and developed skin lesions which resembled graft-versus-host disease (42, 46). Many mice died by the age of 5 weeks (42, 46). Histologic examination of the hearts revealed extensive damage, suggesting that the cause of death was heart failure (46). The BALB/c PD1^-/-^ mice had an increased population of activated CD8^+^ T cells in comparison to WT mice. Interestingly, BALB/c-RAG-2^-/-^PD-1^-/-^ mice did not die but remained healthy, highlighting the role of T cells and/or B cells in disease development (46). Indeed, the hearts of BALB/c PD-1^-/-^ mice had increased depositions of IgG1, IgM, and C3 complement (46). Furthermore, serum from PD-1-deficient mice was enriched with autoantibodies against cardiac troponin I (46, 47).

Deletion of PD-1 from the NOD mouse presented a phenotype consistent with that observed to the two previous strains, with early and robust development of autoimmune activation. NOD-PD-1^-/-^ mice developed diabetes much earlier than in NOD-PD-1^+/+^ mice with early insulitis and increased infiltration of CD4^+^ and CD8^+^ T cells (48). In vitro stimulation of T cells isolated from β-islets of NOD-PD-1^-/-^ mice showed an increased proclivity for IFN-γ secretion.

These studies highlighted the importance of both CTLA-4 and PD-1 in immune homeostasis and their indispensable and non-redundant roles in preventing autoimmunity.

### irAEs mediated by ICI therapies

**Figure 1:**
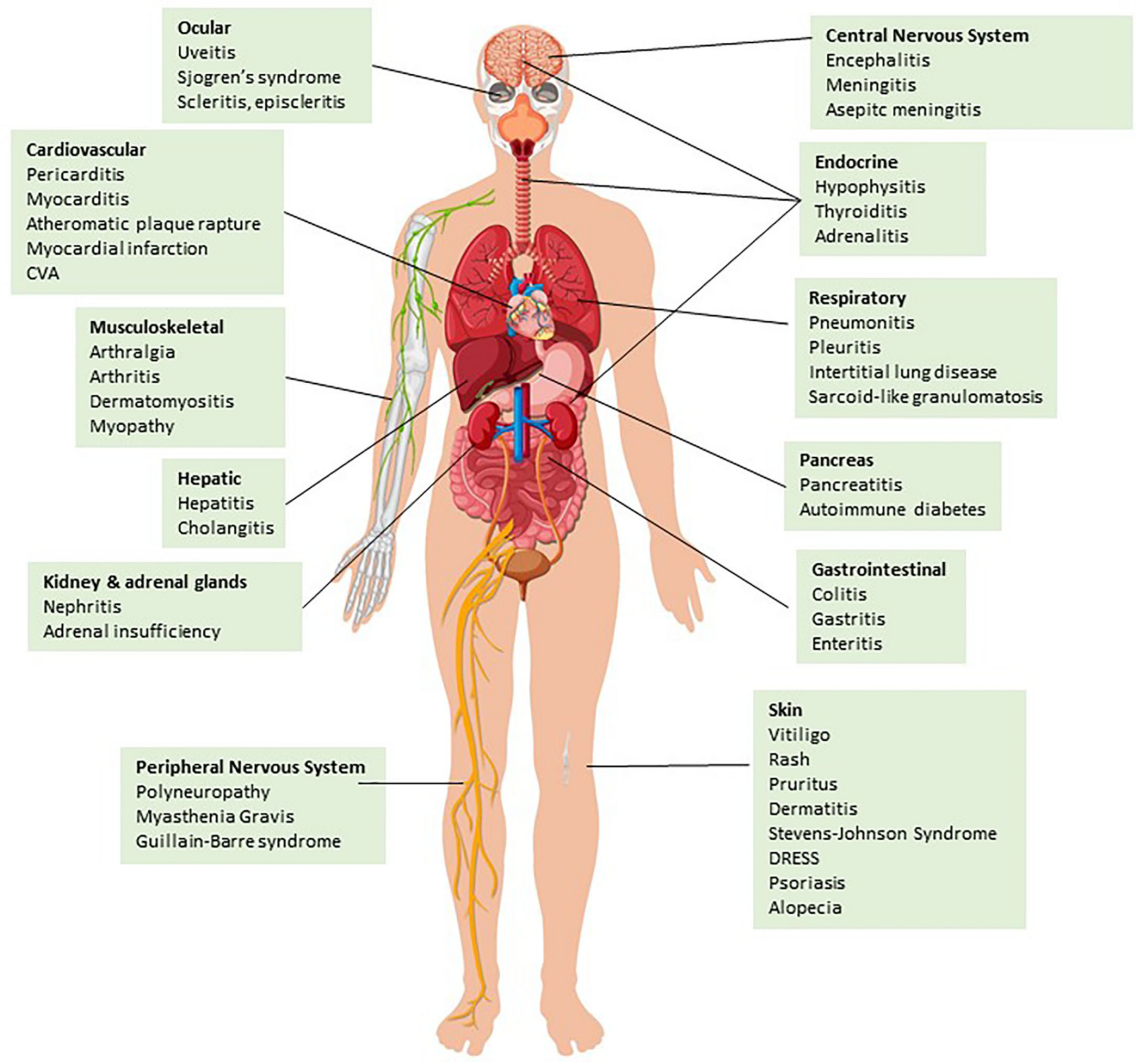
irAEs categorized by organ system. ICI treatment may result in inflammation of various tissues and organ systems leading to well-described irAEs (please see main text for details) Design by Brgfx-Freepik.com.

Based on the physiological properties of CTLA-4 and PD-1 revealed by the extensive studies outlined above, it is anticipated that blockade of these ICs in humans for cancer therapy will inevitably lead to global immune activation leading to autoimmune manifestations of various organ systems (**Figure 1**). Immune checkpoint inhibitors (ICIs) employed for cancer therapy include PD-1 (pembrolizumab, nivolumab, cemiplimab, dostarlimab), PDL-1 (atezolizumab, avelumab, durvalumab), CTLA-4 (ipilimumab, tremelimumab), and the recently approved LAG-3 antibodies (relatlimab). These ICIs are used individually, in combination, or together with chemotherapy (49). ICI therapy differs from chemotherapy in mechanisms of action and side effect profile, which are intimately linked. ICIs aim to increase the activity of the immune system against cancer by breaking tolerance mediated by CTLA-4 and PD-1 in the TME. However, ICI therapy simultaneously activates non-cancer-specific T cell subsets, B cells and myeloid cells which are kept in check by ICs, thereby preventing autoimmunity as outlined in the previous section. Therefore, irAEs are typical side effects of ICIs. Several irAEs resemble features of ADs and may occur during or after discontinuation of ICI treatment. ICI may also trigger the clinical evolution and presentation of previously pre-clinical and unidentified ADs. In addition, irAEs may occur as paraneoplastic syndromes induced by autoantibody production like typical paraneoplastic syndromes in the context of several cancers.

A cross-sectional study showed that 43.63% of patients with cancer in the US were eligible for ICI use (50). Hence, the use of ICIs is becoming increasingly common in various cancers, making the incidence of irAEs a critical problem, since up to 80% of patients treated with ICIs develop irAEs (51). Currently, there is no reliable biomarker predicting or correlating with irAEs, but several ones are under investigation including serum IL-6, IL-17, soluble CTLA-4 (sCTLA-4), absolute lymphocyte count, and tumor mutation burden (52–55). The most frequently reported irAEs include fatigue, pruritus, nausea, rash, diarrhea/colitis, and endocrinopathies (mainly hypophysitis and thyroiditis), the most common serious irAEs are pneumonitis and colitis, whereas myocarditis is the irAE with the highest fatality rate (**Figure 1**) (51, 56).

As the use of ICIs is currently increasing, previously unrecognized rare complications become clinically apparent. Importantly, there is increasing evidence that ICI immunotherapy induces cardiovascular complications to a previously unappreciated level. Myocarditis is a well recognized and extensively studied irAE occurring with higher incidence and severity among patients treated with ipilimumab and nivolumab combination compared to those treated with nivolumab alone (57, 58). Newer studies provide evidence the PD-1/PD-L1 blockade induces a prothrombotic environment leading to vein thromboembolism (59), and to the development or worsening of atherosclerotic cardiovascular disease (CVD) resulting in atherotic plaque rapture and presentation of various clinical pathologies of atherosclerotic CVD including coronary artery disease, myocardial infarction, and ischemic stroke (60). These findings are consistent with enhanced severity of atheromatous cardiac disease observed in experimental models of mice with genetic ablation of ICs or treated with ICIs (61–63). Importantly, recent studies provided evidence that pre-existing autoimmune conditions increase the incidence of CVD after ICI therapy (64). Given the immune-mediated nature of atheromatous disease (65, 66), these observations indicate that CVD is another form of ICI-mediated irAE.

irAEs generally improve with the discontinuation of ICIs with or without administration of immunosuppressive therapy. However, several case reports raise concerns about the potentially irreversible morbidity of irAEs, underlying the role of early diagnosis and proper management of these serious complications. Chronic irAEs may affect up to 40% of patients, with endocrine and rheumatologic manifestations being the more frequent forms of chronic irAEs (67, 68). Guidelines for diagnosis and treatment of irAEs are available and extensively reviewed elsewhere (69–71).

### Pathogenesis of irAEs

**Figure 2:**
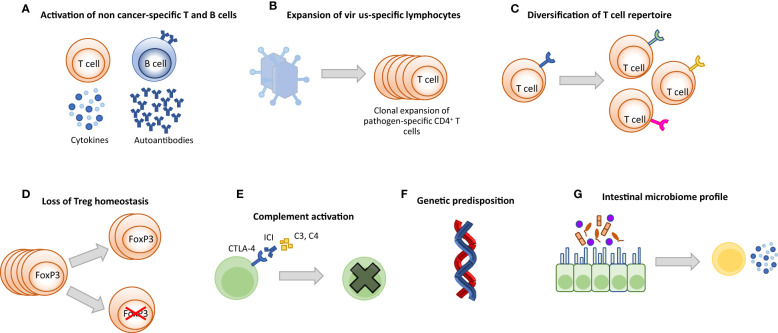
Potential mechanisms driving irAEs. **(A)** Activation of cytotoxic self-reactive T cells causes damage in off-target healthy tissues by extensive production of pro-inflammatory cytokines and/or direct attack. Activated autoreactive B cells may produce *de novo* auto-antibodies or increase the amount of pre-existing auto-antibodies. Antibodies bind to healthy tissues and cause inflammation and damage. **(B)** Clonal proliferation of virus-specific T cells may lead to excessive inflammation and destruction in the relevant organ and may be fatal. **(C)** Expansion of the T cell repertoire may cause T cells to attack off-target healthy tissues. **(D)** ICI treatment can lead to decreased number of FoxP3-expressing Treg cells and reprogramming of Treg cells, resulting in pro-inflammatory behavior. **(E)** Organ-specific expression of ICI targets can induce direct ICI binding followed by complement activation and antibody-mediated inflammation (type II hypersensitivity). **(F)** Genetic polymorphisms such as some HLA allele types, mutations of IC receptors and miRNAs are associated with the development of irAEs. **(G)** Microbiome composition (bacteria, metabolites, etc.) may cause aberrant activation of the immune system and increased production of inflammatory cytokines under ICI treatment.

Although the pathogenesis of irAEs is still not fully understood, several mechanisms have been identified including aberrant T-cell activation, autoantibody production, inflammatory monocyte activation, complement-mediated inflammation, inflammatory cytokines, host-specific factors including microbiome and genetics, and the type of ICI immunotherapy administered (**Figure 2**) (72). Briefly, irAEs can be mechanistically categorized as follows:

#### T cell-mediated

ICI therapy directly causes activation of T cells outside of the TME as it inhibits inhibitory signals that prevent T cell activation against self antigens, leading to autoimmune manifestations from different organs. The increase in unique TCR V-beta CDR3 diversity, a marker of TCR richness, was significantly higher in patients who developed irAE compared to those who did not (73). Several case reports of severe and even lethal manifestations of irAEs provided evidence that clonally expanded self-reactive or virus-reactive T cells are accumulated in the affected tissues, linking self- and pathogen-recognizing T cell clones to lethal toxicity. For example, fulminant myocarditis was associated with infiltration of the myocardium by clonal T cells identical with those infiltrating the tumor and skeletal muscles (74); fatal ICI-associated encephalitis correlated with activation of EBV-specific memory CD4^+^ T cells, whereas ICI-associated hepatitis correlated with activation of CMV-specific T cells (75, 76). CD4^+^ and CD8^+^ T cell enrichment in target tissues was observed in irAEs leading to diabetes, colitis, and thyroiditis (77–79). Subsequent studies provided evidence that the TCR richness of activated CD4^+^ T effector memory cells underlies an overall increase in pretreatment TCR diversity in patients destined to develop severe irAEs. In this context, the magnitude of T cell clonal expansion correlated with the onset time of severe irAEs because patients with a greater magnitude of TCR clonal expansion developed irAEs sooner (80). In addition, Th17.1 cells, known for their role in chronic inflammation, were predominantly increased in bronchoalveolar lavage fluid of patients with ICI-related pneumonitis (81, 82). Lastly, a decrease in the number and an inflammatory reprogramming of Treg are also involved in the pathogenesis of irAEs (83).

#### B cell- and antibody-mediated

The presence of autoantibodies in the pathogenesis of ADs is well known. Although studies have shown that B cells play a role in response to immunotherapy, their role in the development of irAEs is still not fully understood (84). Inherited deficiencies in the B regulatory (Breg) cells, which produce IL-10, have been associated with the development of severe irAEs (85). An increase in CD21^low^ B cell subset and plasmablasts has been identified in patients treated with combination ICIs., whereas circulating B cells were decreased (86). Patients experiencing organ-specific irAEs had a low baseline autoantibody level at baseline, which was significantly increased by 6 weeks after initiation of ICI therapy (87). This finding may suggest that the pathogenesis of irAEs is different from that of classic ADs, where baseline autoantibody levels positively correlate with AD development.

The role of disease-specific autoantibodies in the development of irAEs remains controversial. Sakakida et al. reported that positive anti-nuclear antibody (ANA) titers are associated with a higher risk of ICI-mediated colitis but not classic ANA-associated autoimmune diseases like SLE and scleroderma (88). However, other investigators reported a correlation between the occurrence of organ-specific irAEs and disease-specific autoantibody levels. Osorio et al. found that thyroid dysfunction is associated with anti-thyroid antibodies, whereas Suzuki et al. showed that ICI-related myasthenia gravis is associated with anti-acetylcholine receptor (AChR) antibodies (89, 90). In a retrospective study of patients with non-small cell lung cancer (NSCLC) treated with anti-PD-1, pre-existing ANA, rheumatoid factor (RF), anti-thyroiglobulin and anti-thyroid peroxidase antibodies positivity correlated with the development of irAEs but also with clinical benefit from ICI immunotherapy (91). Notably, these findings were confirmed by a smaller prospective study which determined that the levels of anti-thyroiglobulin and anti-thyroid peroxidase antibodies at baseline were higher in patients who developed thyroiditis after anti-PD-1 blocking immunotherapy (92). Other studies reported that pre-existing AChR antibodies are commonly found in patients who develop irAE-associated myositis and myasthenia gravis (93, 94).

#### Complement-mediated

Since ICIs are monoclonal antibodies, they can bind to off-target tissues and cause inflammation through classical complement cascade activation. This mechanism is thought to play a role in the pathogenesis of anti-CTLA4-mediated hypophysitis by binding abundantly expressed CTLA4 antigen in the pituitary, activating complement, and causing development of complement-dependent antibody-mediated cytotoxicity (CDC) against cells secreting thyrotropin, follicle-stimulating hormone or corticotropin. These cells became the site of deposition of C3d and C4d components and activation of an inflammatory cascade mimicking type II hypersensitivity (95). Autopsy results of patients who developed hypophysitis after treatment with ipilimumab showed that type II hypersensitivity was involved in the early stages of pathogenesis, whereas type IV hypersensitivity caused by infiltration of autoreactive T lymphocytes played a role in the later stages (96).

#### Cytokine-mediated

Since cytokines are mediators of inflammation, they can be used to assess the direction of the systemic immune response. ICI treatment causes a shift of cytokine balance toward an inflammatory profile. For example, elevated circulating IL-17, which is secreted especially by Th17 cells suppressing Treg activity, and IL-2 which enhances activity of cytotoxic CD8^+^ T cells, are associated with the development of irAEs (97, 98). Lim et al. defined a toxicity score (CYTOX) by integrating expression of 11 circulating pro-inflammatory cytokines (G-CSF, GM-CSF, Fractalkine, FGF-2, IFNα2, IL-12p70, IL-1a, IL-1b, IL-1RA, IL-2, IL-13) (98). CYTOX score were significantly higher in patients developed irAEs (98). Certain cytokines have been shown to be involved in the development of specific irAEs. For example, psoriasiform dermatitis has been correlated with increased IL-6 levels, pneumonitis with increased IL-1β, and colitis with increased IL-17 (99–101). In addition, chemokine ligands such as CXCL9, may also potentially contribute to the pathogenesis of irAEs (102).

#### Inflammatory monocyte-mediated

The inflammatory milieu generated by the production of effector cytokines by immune cells which are disinhibited by blockade of ICs during ICI immunotherapy, promotes the differentiation of M1-like monocytes which produce proinflammatory cytokines, such as TNF-α and IL-1β, and convert into macrophages in target tissues. Such inflammatory monocytes can also be generated by direct inhibition of PD-1 mediated signaling during differentiation of myeloid progenitors in response to hematopoietic growth factors produced by cancer and activated T cells (14, 15). Such inflammatory monocytes infiltrate target organs of irAEs such as the lung where they cause severe pneumonitis with granulomatosis (103) and the myocardium, causing life threatening myocarditis (104).

#### Host-specific factors

Factors that regulate immune responses by complex tissue-specific and systemic mechanisms, such as microbiota and gene polymorphisms, have been shown to play important roles in the development of irAEs (105–107). Polymorphism of microRNA146, which is associated with autoimmune diseases and is known to promote a proinflammatory Treg behavior, was shown to correlate with the development of severe irAEs (106, 107). Similarly to conventional ADs, several HLA types have been implicated in the pathogenesis of irAEs. Associations have been identified between HLA DRB1*04: 05 and ICI-induced inflammatory arthritis, HLA-DRB1*11:01 and pruritus, HLA-DQB1*03:01 and colitis (108, 109). However, a recent study reported no association between HLA type and irAE development (110). Thus, further work is required toward this direction.

The gut microbiome has been extensively studied regarding its tentative role in the therapeutic efficacy and toxicity of ICI therapy. *Clostridiales, Ruminococcaceae* or *Faecalibacterium* abundance and high diversity in the microbiota are associated with higher numbers of circulating T cells and responses to anti PD-1 immunotherapy in melanoma patients (111). In a recent prospective study of patients with advanced melanoma who were treated with a combination of anti-PD-1 and anti-CTLA-4 ICIs, the profiling of gut microbiota demonstrated a significantly higher pre-treatment fecal abundance of *Bacteroides intestinalis* in patients with any ≥ grade 3 toxicities, which correlated with upregulation of mucosal IL-1β in biopsy samples and a more diverse peripheral T cell repertoire (105). These findings contrasted with a previous report which had found that pre-treatment faucal abundance of *Bacteroidetes phylum* correlated with resistance to the development of colitis following ICI monotherapy with CTLA-4 blockade (112). It is evident that further work is required to understand the biological relevance and the potential exploitation of the microbiome to enhance the efficacy and limit the toxicity of ICI cancer immunotherapy (113).

#### Type of ICI immunotherapy

A systematic review of 35 randomized clinical trials consisting of 16,485 patients showed that the profile of irAEs depends on the type of immunotherapy. Colitis and hypophysitis were seen more often with CTLA-4 inhibitors, while hypothyroidism, hyperthyroidism, and pneumonitis were more common in PD-1 inhibitors (114). Furthermore, anti-CTLA-4-induced irAEs generally tend to be more frequent and severe (115). Although the differences in irAEs between ICI therapies have not been fully identified, the distinct prevalence of organ-specific irAEs might be related to the unique properties of the targeted receptors. CTLA-4 suppresses T cell responses in the early steps of the activation cascade in lymphoid organs, whereas PD-1 acts in the late stages of the immune response in both lymphoid organs and peripheral tissues (72). CTLA-4 is expressed by T cells and binds to its ligands CD80 or CD86, which are present on professional antigen-presenting cells, while PD-1 is expressed by T cells and many other immune cell types, and its ligand PD-L1 is present on several types of immune cells but also somatic cells and cancer (116–118). Due to the differential expression of CTLA-4 and PD-1, the combined use of PD-1 and CTLA-4 antibodies is associated with an increased risk of irAEs (119, 120), but also prolonged progression-free survival (121, 122).

## irAEs and therapeutic response to ICI immunotherapy

Although the occurrence of irAEs indicates that the immune system has been successfully activated by ICI therapy suggesting that treatment has achieved its goal, there are conflicting data regarding the correlation between irAEs and therapeutic response. Recent studies showed that the development of irAEs in patients with various cancers receiving anti-PD-1 antibody is associated with a significantly higher response rate and increased progression-free survival rate (123, 124). These were consistent with earlier studies on patients treated with CTLA-4 inhibitors, which also showed that the development of irAEs correlated with a high response rate (125, 126). Subsequently, other reports indicated that the occurrence of irAEs in the early stages of treatment was associated with better outcomes, whereas other investigators found no correlation between irAEs and treatment outcome (70). Recently, it was noted that specific irAEs might be selectively associated with therapeutic response and might serve as biomarkers to predict clinical benefit. For example, thyroid, cutaneous and low-grade irAEs, as well as AD flares, are positively correlated with therapeutic outcome, whereas pneumonitis is associated with poor outcomes (127–130).

## Diagnosis of iRAEs and therapeutic intervention

Early diagnosis and determination of the severity of irAEs are crucial to prevent morbidity and mortality. The grading system of irAEs is based on Common Terminology Criteria for Adverse Events (CTCAE) and grades are categorized from 1 to 5 according to the severity. Clinical criteria for diagnosis, grading, and treatment of irAEs have been reviewed extensively elsewhere (69, 70, 131).

Briefly, ICI discontinuation is not necessary in grade 1 irAEs when close monitoring is feasible, whereas holding ICIs should be considered for most grade 2 toxicities. In severe irAEs (grade 3-4), ICI should be discontinued, and steroid should be initiated. If symptoms worsen, steroid-sparing biologic immunosuppressants such as tumor necrosis factor-alpha (TNF-α) inhibitors or IL-6 antagonists should be considered (71). Corticosteroids are the first-line treatment modality and should be administered with dose adjustment according to the severity of symptoms for ≥ 2 grade irAEs (71). If symptoms do not improve with high-dose steroids, TNFα inhibitors can be an option (71). The management of endocrine and rheumatologic irAEs, which often cause chronic morbidity, may differ from therapy for other irAEs. A national multicenter study showed among 117 patients who developed rheumatologic irAEs, 44 patients required disease-modifying anti-rheumatic drugs (DMARD) (132). Hormone replacement is essential in the management of endocrine irAEs. Unlike other systemic irAEs, high-dose steroids are not required because it is unlikely to improve endocrine irAEs caused by damage to endocrine cells.

Cutaneous toxicities have been reported for 30% to 50% of all side effects of ICI therapies (133) and are classified separately. The ASCO committee divided dermatological irAEs into rash/inflammatory dermatitis, bullous dermatoses, and severe cutaneous adverse effects (SCARs) including Stevens-Johnson Syndrome (SJS), toxic epidermal necrolysis (TEN) and drug rash with eosinophilia and systemic symptoms (DRESS). Treatments for these skin manifestations depend on grading. Inflammatory reaction that affects the quality of life or is grade ≥ 2 should be considered as an indication to hold the ICI and monitor patients weekly for improvement. Blistering lesions that extend more than 10-30% of body surface area (BSA) or grade ≥ 2 should mandate withdrawing the ICI therapy and urge dermatological work up. Development of blistering lesions covering ≥ 30% of BSA mandates permanent discontinuation of ICI. SCARs, including SJS, TEN, acute generalized exanthematous pustulosis (AGEP), and DRESS/drug-induced hypersensitivity syndrome (DiHS) should mandate immediate discontinuation of ICI therapy, regardless of the grade, and close follow-up. Dermatology consultation is critical for appraising the risks and benefits of withdrawing or rechallenging with ICI immunotherapy. Systemic steroids are used in grade 3 of rash and inflammatory lesions. Blistering that affects the quality of life and meets the criteria for grade 2 toxicity with 10-30% BSA involvement should be managed with high potency topical steroid and consideration for systemic steroids therapy. For SCARs, prednisolone or equivalent agent should be initiated in grade 2, which is a morbilliform exanthem at 10-30% BSA together with a systemic symptom or lymphadenopathy.

A pigmented lesion which is a vitiligo-like depigmentation (VLD) has been reported exclusively in melanoma patients. To study the cumulative incidence of VLD, 137 studies conducted in patients with stage III or IV melanoma were recruited and showed an overall cumulative incidence of VLD at 3.4% (95% CI, 2.5% to 4.5%). The development of VLD significantly correlated with response to ICI therapy. Patients who developed VLD during ICI treatment for melanoma had a 2-fold lower risk of disease progression and a 4-fold lower risk of death, progression-free survival (hazard ratio [HR], 0.51; 95% CI, 0.32 to 0.82; p<0.005) and overall survival (HR, 0.25; 95% CI, 0.10 to 0.61; p<0.003) (134). Similarly, in a retrospective analysis of 148 patients who received nivolumab plus peptide vaccine or nivolumab alone, a significantly higher overall survival was observed in patients who developed rash (hazard ratio [HR], 0.423; 95% CI, 0.243 to 0.735; p=0.001) and VLD (HR, 0.184; 95% CI, 0.036 to 0.94; p=0.012 (31). VLD occurring after ICI therapy in melanoma illustrates that melanoma-specific T cells activated after ICI also recognize shared antigens of normal melanocytes and their presence might imply a better response to immunotherapy and a favorable prognosis (135).

The impact of high-dose steroid therapy on ICI efficacy and clinical outcome remains controversial. Some studies showed that high-dose corticosteroids are associated with poor outcomes (123, 136, 137) warning for cautious consideration of all parameters when the use of high-dose steroids is a tentative choice. Notably, a recent study revealed that only 47% of irAEs were managed according to guidelines, whereas 38.8% of irAEs had no documented management (138).

## The role of ICIs in patients with cancer and pre-existing ADs

In the modern era of increasing incidence of cancer and ADs, up to a quarter of patients have both diseases at the same time (139). Thus, there are many cancer patients with pre-existing ADs who might be benefit from the use of ICI. Treatment of patients with pre-existing AD raises concerns for potential flair-ups due to their pre-activated immune system. As a result, clinical trials preclude enrollment of these patients. However, this raises important questions regarding the efficacy of ICI treatment in these patients and the potential complications such as flares or *de novo* irAEs post-ICI treatment if patients were administered immunosuppression for their AD. As currently there is increasing interest regarding eligibility of patients with pre-existing AD for ICI therapies, several retrospective studies and meta-analyses have begun assessing outcomes and toxicities in this cohort of patients (140–155). Key studies are summarized in **Table 1**.

**Table 1:** Summary of key studies using ICI in patients with cancer and pre-existing autoimmune diseases.

Main pre-existing AD	ICI used	Number of patients	Flare of pre-existing AD after ICIs	New irAEs	Management	Reference
Psoriasis, MS, RA, SLE, IBD, sarcoidosis, thyroiditis	Anti-CTLA-4	30	27%	33%	Steroid, SSA (infliximab)	(141)
PMR, SLE, RA, psoriasis, IBD, SS	Anti-PD-1	52	38%	29%	Steroid, SSA, IVIG, ICI discontinuation 11%	(142)
RA, vitiligo, thyroiditis, SS	Anti-PD-1	45	24%	44%	Steroid, ICI discontinuation 25%	(143)
Psoriasis, RA, PMR, IBD, thyroiditis	Anti-PD-1 or anti-PD-L1	56	23%	38%	Steroid, ICI discontinuation 14%	(144)
RA, psoriasis, IBD, thyroiditis	Anti-CTLA-4	41	29%	29%	Steroid, SSA (infliximab, hydroxychloroquine, sulfasalazine)	(145)
RA, thyroiditis, psoriasis, IBD, PMR, SLE	Anti-PD-1	85	47%	66%	ICI discontinuation 7%	(146)
RA, psoriasis, IBD, SLE, PMR	All ICIs (anti-CTLA-4, anti-PD-1, anti-PD-L1)	112	47%	42%	Steroid, SSA (azathioprine, methotrexate, TNF inhibitor), IVIG, ICI discontinuation 21%	(147)
RA	All	22	55%	32%	Steroid, ICI discontinuation 23%	(148)
RA, PMR, SLE, psoriasis, sarcoidosis, IBD, thyroiditis	All	106	36%	38%	Steroid, SSA (methotrexate, rituximab, infliximab), ICI discontinuation 20%	(149)
RA, thyroiditis, psoriasis	Anti-PD-L1	35	11%	46%	Steroid, ICI discontinuation 9%	(150)
RA, type 1 DM, atrophic gastritis	All	106	N/A	58%	N/A	(151)
RA, SLE, SSc, IBD, sarcoidosis, hyperthyroid-ism, hypothyroid-ism	All	415	N/A	Comb. 44%, anti-CTLA-4 30%, anti-PD-1 17%	Steroid, SSA (TNF inhibitor), ICI discontinuation 17%	(152)
SLE, RA, psoriasis, IBD, sarcoidosis	Anti-PD-1	47	26%	N/A	Steroid, ICI discontinuation 11%	(153)
RA, hypothyroidism, psoriasis	Anti-PD-1, anti-PD-L1 or combination	63	31%	62%	Steroid, SSA	(154)
RA, PMR, SLE, IBD, psoriasis, scleroderma, sarcoidosis	Anti-CTLA-4, anti-PD-1 or combination	74	N/A	50% mild, 37% severe	N/A	(140)
IBD, RA, MS, hypothyroidism, microscopic colitis	Anti-CTLA-4, anti-PD-1, anti-PD-L1 or combination	197	14.7%	25.3%	Steroid, infiximab and vedolizumab	(155)

IBD, inflammatory bowel disease; ICI, immune-checkpoint inhibitor; IVIG, intravenous immunoglobulins; MS, multiple sclerosis; N/A, not applicable; PMR, polymyalgia rheumatica; SS: Sjögren’s syndrome; RA, rheumatoid arthritis; SLE, systemic lupus erythematosus; SSA, steroid-sparing agent.

Psoriasis and psoriatic arthritis patients have the highest risk of developing flare or *de novo* irAEs after ICI treatment (147, 156). Patients with rheumatoid arthritis (RA) experience flares of their disease at approximately 60% (147, 157). A retrospective study showed that there is no association between the presence of pre-existing AD and the development of irAEs, the number of irAEs, and their severity (154). In a study on melanoma and NSCLC patients, there was no association between overall survival and the presence of AD, however, a later study showed that patients with pre-existing AD had an increased overall survival compared to patients without AD (157). Other studies have shown that the development of irAEs is more prevalent in patients with a pre-existing AD (157, 158). Gulati et al. showed that melanoma patients with pre-existing AD had an increased progression-free survival and development of irAEs when treated with ICI (155). Patients with pre-existing AD who received ICI and survived for more than 1 year developed new-onset chronic kidney disease and experienced a rapid drop in glomerular filtration rate, which is used as a marker for kidney function (159).

Development of *de novo* irAEs is more common in patients with pre-existing AD after administration of ICI. Patients with RA have a higher frequency of all-grade irAEs and severe irAEs (157). A separate study found that only 14.7% of patients had an AD flare while 25.3% developed a new irAEs (140). Most of the patients in this study had quiescent AD and 9.6% were receiving steroid treatment at the time ICI therapy was initiated. Patients with gastrointestinal and rheumatologic AD had the most frequent flare-ups after ICI therapy, whereas patients with Hashimoto thyroiditis and neurologic AD developed mostly new irAEs. Studies with over 100 patients with pre-existing AD who received ICI treatment showed that 25-50% developed flares of their disease, whereas about 40% developed *de novo* irAEs (160). Abdel-Wahad et al. reported that no differences were observed in the presentation of irAEs between active and inactive AD, however, Wu et al. showed that patients with inactive AD presented high-grade irAEs more frequently compared to patients with active AD (156, 161). Moreover, patients on immunosuppression at the initiation of ICI showed lower rates for irAEs, especially high-grade, compared to those who were not on immunosuppression (156, 161).

Disease flares were increased in patients receiving anti-PD-1/PD-L1 treatment, whereas *de novo* irAEs were observed more often when patients received anti-CTLA-4 therapy (156). In a small study where anti-PD-L1 and anti-PD-1 treatment was administered to cancer patients with systemic sclerosis, 24% of patients experienced flares and almost 60% developed irAEs, with only 6% developing grade 3-4 irAEs (162). Patients with AD who received anti-PD-1 treatment developed irAEs of any grade at an increased level compared to those with no pre-existing AD (163). In a multicenter study, 33% of patients with advanced melanoma with AD which received a combination of anti-CTLA-4 and anti-PD-1 treatment developed a flare, most commonly rheumatic or gastrointestinal irAEs (164). Interestingly, patients without immunosuppression before the administration of ICI had an increased overall survival compared to those who were on immunosuppression. Furthermore, patients on immunosuppression had an increased risk of developing flares.

A novel retrospective study showed that patients who received ICI and had a pre-existing AD (most common being RA, psoriasis, and polymyalgia rheumatica) developed cardiovascular irAEs more often than patients without AD (64) indicating that such patients should be actively monitored for cardiovascular toxicities. However, more than two-thirds of patients receiving immunosuppression for their AD before ICI administration developed other non-cardiovascular-related adverse effects.

Patients without pre-existing AD had increased association of developing rheumatic-irAEs if they were positive for rheumatoid factor before getting administered ICI (165). Interestingly, other studies reported that the presence of rheumatic-irAEs and ICI-arthritis resulted in increased overall survival and only 35% of patients with pre-exisiting AD developed flare-ups. ICI-induced thyroiditis has been shown to be associated with improved overall survival and progression-free survival compared to patients who did not develop thyroiditis (166, 167). The association between overall survival and thyroiditis varied between different tumors, however, it was related strongly with lung cancer.

## Conclusions and perspectives

There is a substantial number of patients with AD and cancer. Because such patients were excluded from clinical trials, only limited data are available regarding the efficacy and safety of ICIs in this large patient population. The limited number of studies available have shown that using immunotherapy in patients with ADs causes an increased risk of irAEs and AD flares, but irAEs are mostly transient and manageable and rarely mandate treatment discontinuation and life-threatening complications. The use of ICIs in patients with AD is often equally safe as in patients without AD. Hence, pre-existent AD is not an absolute contraindication for the use of ICIs. The lack of a validated predictive biomarker requires a careful evaluation for early recognition of irAEs.

The less frequent and milder adverse effect profile of PD-1 inhibitors compared to CTLA-4 inhibitors suggests that PD-1 inhibitors might be considered as the immunotherapy of choice for cancer patients with pre-existing ADs. In cases that develop irAEs with or without pre-existing ADs a multidisciplinary approach and close monitoring are mandatory. Since the pathogenesis of organ-specific irAEs is unique, approaches of personalized therapy will be required to avoid non-specific immunosuppression and preserve the therapeutic benefit of ICI immunotherapy.

## Author contributions

BI wrote the main body of the manuscript and prepared figures. KA wrote main sections of the manuscript. CC wrote sections of the manuscript. SY wrote sections of the manuscript. VB wrote sections of the manuscript and supervised all the co-authors in the preparation of their contributions. All authors reviewed the manuscript and agree with its entire content. All authors contributed to the article.
